# Therapeutic Potential of Apocynin: A Promising Antioxidant Strategy for Acute Kidney Injury

**DOI:** 10.3390/antiox14081025

**Published:** 2025-08-21

**Authors:** Jelena Nesovic Ostojic, Sanjin Kovacevic, Silvio R. De Luka, Milan Ivanov, Aleksandra Nenadovic, Andrija Vukovic

**Affiliations:** 1 Department of Pathological Physiology, Faculty of Medicine, University of Belgrade, 11000 Belgrade, Serbia; sanjin.kovacevic@med.bg.ac.rs (S.K.); silvio.de-luka@med.bg.ac.rs (S.R.D.L.); aleksandra.nenadovic@med.bg.ac.rs (A.N.); andrija.vukovic@med.bg.ac.rs (A.V.); 2 Institute for Medical Research, Department of Cardiovascular Physiology, National Institute of Republic of Serbia, University of Belgrade, 11000 Belgrade, Serbia; ivmilan@imi.bg.ac.rs

**Keywords:** acute kidney injury, oxidative stress, NOX, apocynin, acetovanillone, ischemia–reperfusion injury, nephrotoxic-induced AKI

## Abstract

Acute kidney injury (AKI) is characterized by a sudden rise in serum creatinine levels, a reduction in urine output, or both. Despite its frequent occurrence in clinical settings, AKI remains poorly understood from a pathophysiological standpoint. As a result, management primarily relies on supportive care rather than targeted treatments. Emerging evidence underscores the pivotal role of oxidative stress in both the initiation and progression of AKI, thereby identifying it as a potential therapeutic target. This review aims to comprehensively examine the pharmacological effects and underlying mechanisms of apocynin (APO) in the context of AKI, with a particular focus on ischemia–reperfusion injury (IRI) and nephrotoxic-induced AKI. Experimental preclinical studies have consistently demonstrated that APO offers protective effects primarily through its inhibition of NADPH oxidase-mediated oxidative stress. In renal IRI and drug-induced nephrotoxicity models, APO has been shown to attenuate oxidative damage, reduce inflammatory responses, and preserve renal structure and function. These results suggest that it may serve as an effective treatment for reducing kidney damage caused by acute ischemia or exposure to nephrotoxic agents. Although the results are encouraging, further investigation is required to establish the optimal dosing strategy and treatment protocol, as well as to confirm the translational relevance of these findings in human clinical settings.

## 1. Introduction

Acute kidney injury (AKI) is characterized by a sudden rise in serum creatinine levels, a reduction in urine output, or both [1]. The global incidence of AKI is rising, with an estimated 13.3 million cases and 1.7 million deaths occurring each year worldwide [2]. According to data from the multinational AKI-EPI study, which included diverse patient populations across several countries, AKI affects approximately 10–15% of hospitalized patients and occurs in more than 50% of those admitted to intensive care units [1,3]. Despite its frequent occurrence in clinical settings, AKI remains poorly understood from a pathophysiological standpoint. As a result, management primarily relies on supportive care rather than targeted treatments. The most common etiology of acute kidney injury (AKI) is renal hypoperfusion, typically due to diminished renal blood flow [4,5,6]. Furthermore, therapeutic agents, including antibiotics, analgetics, chemotherapeutics, and immunosuppressants, are well-established inducers of nephrotoxicity through various pathophysiological mechanisms.

### 1.1. Oxidative Stress in Acute Kidney Injury

Oxidative stress represents one of the most important components in this pathological mechanism of AKI, being marked by circuitous and interdependent pathways leading to organ response as well as damage [7]. Increasing evidence supports the involvement of oxidative stress in both the onset and progression of AKI [8], highlighting it as a potential target for therapeutic intervention. Oxidative damage, manifested as nucleic acid modifications, lipid peroxidation, and protein structural alterations, contributes significantly to renal cellular injury and dysfunction. Key mediators of this damage, including reactive oxygen species (ROS), reactive nitrogen species (RNS), and free radicals (FRs), are intricately involved in the pathophysiology of AKI [9].

Ischemia and subsequent reperfusion are significant triggers of oxidative stress, with mitochondria serving as the main source of ROS in this context [10,11]. In ischemia–reperfusion injury (IRI), the reperfusion phase is particularly critical, as it is when the most severe tissue damage typically occurs. One of the earliest events following reperfusion is a rapid surge in mitochondrial superoxide anion production, which is released into the cell and acts as a key trigger for the subsequent pathological processes [12,13]. During ischemic injury, various ROS, including hydroxyl radicals, peroxynitrite, and hypochlorous acid are produced [8]. Additionally, the early inflammatory response in IRI is characterized by the recruitment of neutrophils, which are both attracted by ROS and are significant producers of ROS themselves [14]. These ROS, in turn, affect vascular tone by promoting vasoconstriction and increasing renal vascular resistance [15,16]. Research involving critically ill and septic patients with AKI has demonstrated that increased levels of circulating protein and lipid oxidation products are associated with elevated proinflammatory and pro-oxidative mediators and cytokines [17]. In these patients, AKI and oxidative stress are closely linked through a reciprocal pathogenic mechanism, whereby oxidative stress not only arises as a consequence of AKI but also amplifies renal injury, thereby sustaining the cycle of damage [5].

Among the primary forms of drug-induced AKI, acute interstitial nephritis (AIN) and acute tubular necrosis (ATN) are the most commonly observed patterns of renal damage [18,19]. At the cellular level, oxidative stress represents a key pathogenic mechanism, significantly contributing to the development and progression of ATN [19]. In ATN, renal proximal tubular epithelial cells experience a complex sequence of events marked by a progressive loss of polarity and cytoskeletal integrity, followed by necrosis and apoptosis. The resulting necrosis then triggers an inflammatory response [19]. Proximal tubular toxicity arises from direct nephrotoxic insults, including mitochondrial dysfunction, inhibition of lysosomal hydrolases, phospholipid damage, and elevated intracellular calcium levels. These disturbances promote the generation of ROS, resulting in harmful oxidative stress [19]. Simultaneously, the levels of essential antioxidant enzymes, including superoxide dismutase (SOD), catalase, and glutathione reductase, decline. This reduction has been observed in renal tissues following both ischemic injury and exposure to nephrotoxic agents [20].

Ongoing research seeks to clarify the molecular mechanisms underlying renal injury, but the heterogeneity of etiologies such as ischemia–reperfusion injury, nephrotoxic insults, and the influence of comorbidities complicates definitive conclusions [9]. While experimental and clinical studies have investigated various pharmacological interventions, outcomes remain inconclusive. A more comprehensive understanding of these complex pathophysiological processes is essential for developing effective preventive strategies [21]. Current therapeutic strategies aimed at reducing ROS generation in these pathological conditions are being extensively investigated and typically involve natural antioxidant enzymes, synthetic compounds with antioxidant properties, antioxidant mimetics, and various vitamins [22].

### 1.2. Apocynin as an Antioxidant

Apocynin (APO), also known as acetovanillone, is a non-toxic natural compound extracted from the roots of *Picrorhiza kurroa*, a medicinal plant native to the Alpine Himalayan region [23]. The Nicotinamide Adenine Dinucleotide Phosphate (NADPH) Oxidase (NOX) family, a key source of ROS in eukaryotic cells, consists of seven isoforms that play vital roles in a range of human physiological processes [24,25]. Apocynin is a widely studied NOX inhibitor, [24] although it functions as a prodrug with antioxidant properties rather than acting directly on NOX enzymes [26]. APO exerts its inhibitory effect on NADPH oxidase by interacting with critical thiol groups found in its subunits. This interaction specifically interferes with the translocation of the cytosolic subunit p47^phox^ to the cell membrane, an essential step required for the full assembly and activation of the NADPH oxidase enzyme complex [27,28]. Actually, upon activation by peroxidases, APO generates a radical that can form adducts with p47^phox^ [29] or create multimers, which are believed to inhibit NOX through mechanisms that are not yet fully understood [30,31]. However, results from studies using APO should be interpreted with caution, [24] as its activation can also lead to oxidative stress and depletion of glutathione [31], particularly in cells with low NOX activity but significant peroxidase expression conditions, under which ROS levels may actually increase [32]. In addition to its role as a NOX inhibitor, APO has demonstrated NOX-independent effects, such as the suppression of agonist-induced platelet activation [33] and the inhibition of Rho kinase activity [34]. It has been explored as a potential therapeutic agent across a variety of pathological conditions, including cancer, hypertension, atherosclerosis, and ischemia/reperfusion injury [35].

**Figure 1:**
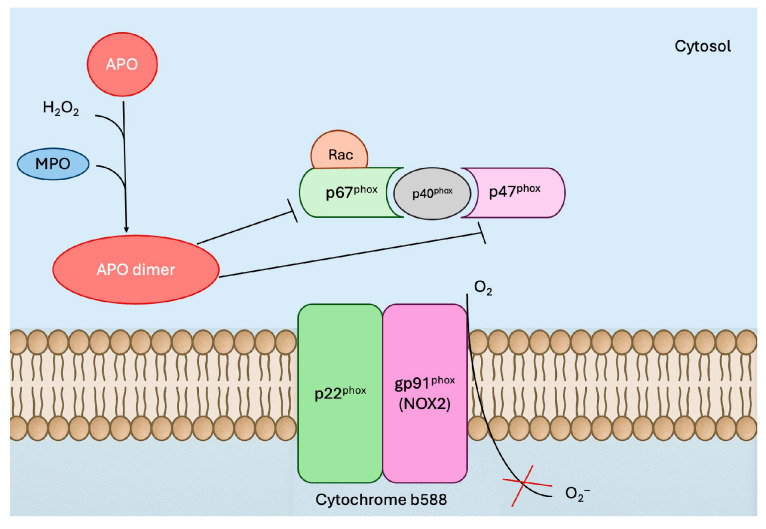
Mechanism of action of apocynin (APO) in leukocytes. APO prevents the assembly of the active NOX2 complex rather than directly inhibiting its catalytic activity. In the presence of myeloperoxidase (MPO) and H_2_O_2_, APO is oxidized to dimerized forms (APO dimers), which block the translocation of cytosolic p47^phox^ and p67^phox^ to the membrane. This prevents their interaction with membrane-bound p22^phox^ and catalytic gp91^phox^, thereby inhibiting NOX2 assembly and subsequent electron transfer from NADPH to O_2_ to form superoxide (O_2_^−^). APO is more effective in MPO-containing leukocytes (granulocytes and monocytes) and has little effect once the NOX2 complex is already assembled.

Apocynin has been shown to interfere with the detection of ROS in assays specific to hydrogen peroxide and hydroxyl radicals. Notably, it directly disrupts the detection of peroxides, but not superoxide, when generated by systems such as xanthine/xanthine oxidase or nonenzymatic reactions. In leukocytes, APO functions as a prodrug that is activated by myeloperoxidase, leading to the formation of APO dimers (Figure 1). However, endothelial and smooth muscle cells lack this activation capability, as they do not produce these dimers, and therefore cannot activate APO [26]. Nevertheless, the cellular effects of APO remain incompletely understood and require further investigation.

This review aims to comprehensively examine the pharmacological effects and underlying mechanisms of APO in the context of AKI, with a particular focus on IRI and nephrotoxic-induced AKI. Specifically, it explores APO’s role as a modulator of oxidative stress and inflammation, evaluates its potential renoprotective properties in experimental models of AKI, and discusses the limitations and inconsistencies in current research. By synthesizing these insights, this review seeks to clarify APO’s therapeutic potential in AKI and guide future research directions.

## 2. Materials and Methods

**Figure 2:**
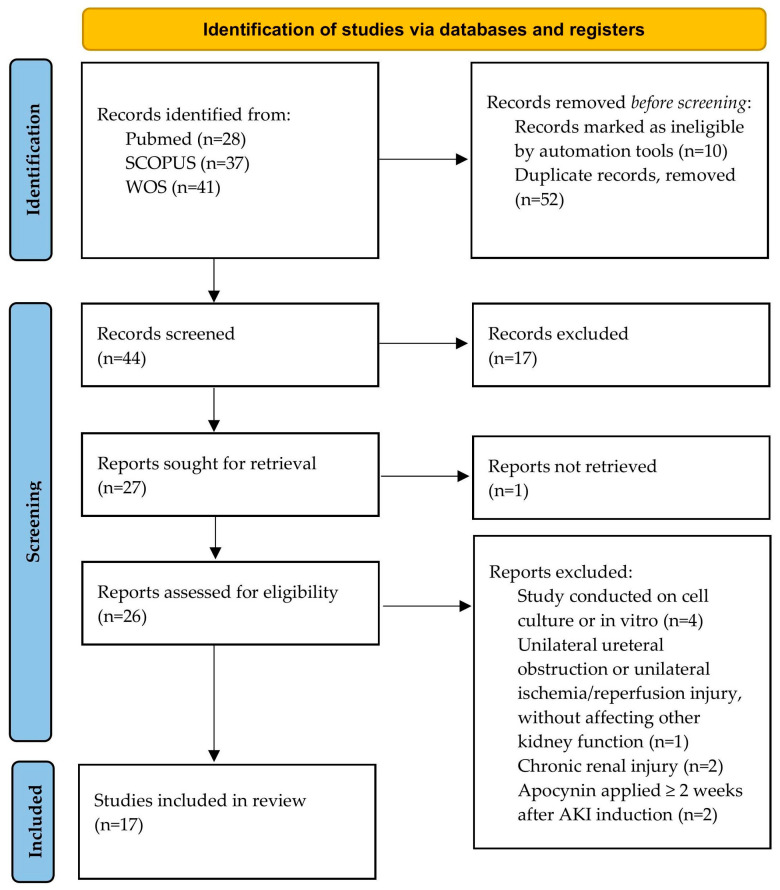
PRISMA flow diagram for the studies selection [36].

The literature review was conducted according to PRISMA guidelines [36]. In the publication search, three databases were used: Pubmed, Scopus, and Web of Science. The literature search was conducted by two independent reviewers during April 2025, using the following keywords: acute kidney injury or acute renal failure or nephrotoxicity and apocynin or acetovanillone. Included articles were original articles conducted on different experimental animal models of AKI, affecting both kidneys, with systemic involvement, in last 20 years, written in English, and that demonstrated the effects of APO applied before, during, or immediately after experimental procedures on kidney function, morphology, and cellular (patho)physiology. Information from the selected articles was extracted and categorized based on the findings, and then summarized to analyze and compare the respective results (Figure 2). This review was registered on the Open Science Framework Registries (OSF)—https://doi.org/10.17605/OSF.IO/2GJ4B.

## 3. Results

Eight studies investigated the effects of APO on postischemic AKI [37,38,39,40,41,42,43,44]. Three studies employed bilateral renal IRI [38,40,42], while five utilized unilateral IRI with contralateral nephrectomy [37,39,41,43,44]. The duration of ischemia varied across studies, ranging from 15 to 60 min [37,38,39,40,41,42,43,44]. Most studies assessed outcomes 24 to 48 h after reperfusion [37,38,39,40,41,43,44]; however, one study evaluated effects four weeks post-procedure [42]. Animal models included Sprague–Dawley rats [38,39,40], Wistar albino rats [37,42], spontaneously hypertensive rats [41,43], and C57BL/6 mice [44]. Apocynin was administered in various protocols, as a single dose, intraperitoneally, 30 or 60 min before ischemia (pre-treatment) [37,39,40], or 5 or 30 min before reperfusion (treatment) [37,38,41,43]. In one study, APO was administered 24 h before and 24 h after AKI induction as acute treatment and 24 h after AKI induction for 7 days as chronic treatment [42]. Another study employed a regimen of 15 min before ischemia (intravenously), immediately after ischemia, and 120 min after ischemia (intraperitoneally) [44]. Applied doses ranged from 20 to 100 mg/kg in rats (2–8) and 10 mg/kg in mice [44]. The main findings from these studies are summarized in Table 1.

Nine studies investigated the effects of APO on AKI induced by various etiological factors [45,46,47,48,49,50,51,52,53]. Three studies employed cisplatin-induced nephrotoxicity [45,46,47], while others utilized gentamicin [48], cyclosporine A [49], contrast [50], and acrylamide [51]. Additionally, two studies examined the effects in models of orthopedic trauma [52] and acute hypertriglyceridemic pancreatitis-induced AKI [53]. Most studies assessed outcomes 1 to 14 days after the experimental procedure, including single intraperitoneal cisplatin administration, single intravenous iomeprol application, trauma induction, and acute hypertriglyceridemic pancreatitis induction [45,46,47,50,52,53]. However, three studies evaluated effects 7 to 14 days after APO co-administration with gentamicin, cyclosporine A, or acrylamide [48,49,51]. Animal models included Wistar albino rats [45,48,51], diabetic Wistar albino rats [50], Wistar-Kyoto rats [49], obese Zucker rats [52], Sprague–Dawley rats [53], and C57BL/6 mice [46,47]. Apocynin was administered in various protocols: as a single dose, intraperitoneally, intravenously, or subcutaneously, 30 min to 24 h before experimental procedure, as a pre-treatment [47,50,53]; and immediately after experimental procedure as a treatment [52], or as a combined pre-treatment and treatment 7 days before and 3 days after [45], 2 h before and 2 days after [46], and 7 days before and 7 days during experimental procedure [48]. Two studies examined apocynin applied along with harmful substances, such as cyclosporine A for 14 days [49] and acrylamide for 10 days [51]. Applied doses ranged from 10 to 100 mg/kg for intraperitoneal administration [46,47,48], 5 mg/kg for intravenous administration [42], 50 mg/kg for subcutaneous [53], and 100 mg/kg for oral administration [51]. Apocynin was dissolved in drinking water ad libitum in three studies: 2 g/L [45], 2,5 mmol/L [47], and 2 mmol/L [52]. The main findings from these studies are summarized in Table 2.

## 4. Discussion

### 4.1. The Effects of Apocynin in Ischemia–Reperfusion Acute Kidney Injury

The presented preclinical studies that investigated the effects of APO in ischemia–reperfusion acute kidney injury utilize a variety of experimental protocols, reflecting differences in both the timing and method of drug administration. Specifically, APO has been applied either as a preconditioning agent prior to ischemia or as a therapeutic intervention during or after reperfusion. Additionally, studies have employed a wide range of dosages, from single intraperitoneal injections (e.g., 10–50 mg/kg) to oral administration over several weeks (e.g., 100 mg/kg/day) and have varied considerably in ischemia and reperfusion durations, from brief 15 min ischemic periods to more prolonged ischemia followed by short- or long-term reperfusion. In all these studies, APO has shown protective effects by reducing oxidative damage, modulating inflammatory responses, and preserving renal histology and function. Its antioxidant activity, primarily through suppression of NADPH oxidase-mediated superoxide production, may play a critical role in attenuating tubular epithelial cell injury and leukocyte infiltration during reperfusion. Reversz et al., 2024. [44] suggest that NADPH oxidases play a significant role in the pathogenesis of mild renal IRI. In cases of severe acute ischemia, the extent of kidney damage appears to surpass the protective capacity of anti-NOX interventions, indicating that such treatments may be ineffective under more severe ischemic conditions [44]. NOX2 originates from two main sources: locally within renal parenchymal cells and from phagocytic cells, predominantly infiltrating neutrophils. Neutrophil infiltration significantly increased following IRI, as evidenced by a progressive rise in MPO immunohistochemical staining starting at 3 h post-reperfusion and persisting for up to 48 h in the affected kidney [54]. Reversz et al. [44] showed that the mRNA expression levels of renal NOX2, NOX4, and p22^phox^ peaked at 3 h after reperfusion. Additionally, a second increase in NOX2 expression was detected at 24 h, coinciding with the peak of neutrophil infiltration in the injured kidney. These findings support the presence of a biphasic oxidative stress response in renal IRI. The initial phase is triggered by a rapid post-ischemic upregulation of local NOX enzymes, leading to early oxidative damage, and a secondary phase, characterized by neutrophil infiltration into the renal interstitium, where NOX2-driven respiratory bursts further exacerbate tissue injury. Moreover, in cases of severe kidney injury caused by prolonged ischemia, oxidative stress likely contributes only marginally to further damage, as other multiple harmful mechanisms are simultaneously activated [44]. On the other hand, Altintas and colleagues [37] evaluated the protective effects of APO in renal IRI, following a 1 h ischemic period. Apocynin administration led to varying degrees of improvement across treatment groups. Histological improvement was most pronounced when APO was administered during ischemia, while significant reductions in MDA and MPO levels, key biochemical markers of oxidative and inflammatory injury, were observed when it was given prior to ischemia. They found beneficial effects of APO in severe ischemia, contrary to Reversz et al. [44], but the applied dose of APO was higher at 20 mg/kg i.p. instead of 10 mg/kg i.p. used by Reversz et al. Altintas and coworkers showed that APO reduced tubular necrosis, apoptosis, and glomerular damage, with more pronounced improvement observed when it was administered during the ischemic period [37]. On the other hand, APO may also promote apoptotic cell death as an alternative protective mechanism, potentially limiting the more detrimental effects of necrotic cell death associated with IRI [55].

As previously mentioned, post-ischemic AKI is accompanied by a strong inflammatory response, primarily mediated by the secretion of various proinflammatory cytokines and chemokines by damaged tubular cells, which is further amplified during the reperfusion phase through secretion by inflammatory cells. Selected studies suggest that, in addition to its antioxidant role, APO also exhibits strong anti-inflammatory properties, primarily reflected in the reduction of TNF-α TLR 4, NF-κB, and IL-6 levels, as well as an increase in IL-10 [38,40,44]. In addition, Revesz showed reduced F4/80 mRNA levels, indicating decreased infiltration by macrophages [44].

The fact that preconditioning with APO may be protective in ischemic reperfusion AKI was confirmed in a study performed by Hu and Wu (2017) [40], too. Their findings indicate that APO may offer preventive effects against renal IRI in rats by modulating zinc levels and metallothionein expression associated with oxidative stress. The demonstrated protective effects of APO as a preconditioning agent are particularly noteworthy given its non-toxic nature [56], favorable safety profile [57,58], and suitability for long-term use. These attributes enhance its therapeutic appeal, as APO not only effectively reduces oxidative stress and inflammation but also does so without introducing adverse effects.

The introduced preclinical studies collectively confirm that APO may improve renal function in IRI, as evidenced by reductions in serum creatinine and blood urea nitrogen (BUN) levels [37,38,39,40,41], as well as improvements in estimated glomerular filtration rate (eGFR), [43]. Kovacevic et al. (2020) [41] provoked 45 min lasting ischemia and showed that i.v. APO injection 5 min before reperfusion improved renal hemodynamics by increasing renal blood flow (RBF) and decreasing renal vascular resistance (RVR). Reactive oxygen species directly influence the reactivity of renal blood vessels, with elevated ROS levels linked to increased RVR [59]. In many tissues, including the kidneys, particularly during AKI, NADPH oxidase is the primary source of ROS [47]. Lima et al. [42] investigated the protective effects of APO, a NADPH oxidase inhibitor, on renal IRI in rats, with a particular focus on ATP-dependent sodium (Na^+^) transport in proximal tubules and intrarenal RAAS activation. Rats subjected to bilateral IRI were monitored for four weeks and treated with APO either continuously (100 mg/kg/day via drinking water) or briefly (24 h before and after IRI). APO treatment effectively prevented the long-lasting impairments in Na^+^ transport and dysregulation of RAAS typically induced by IRI, changes that are implicated in the development of post-ischemic hypertension. These protective effects were attributed to APO’s ability to inhibit NADPH oxidase-mediated oxidative stress. Angiotensin II is known to activate NADPH oxidase, initiating pathways involved in inflammation, apoptosis, and vascular dysfunction [60]. By inhibiting this enzyme, APO may help restore RBF early after injury by modulating RVR [61], thereby preventing the rise in blood pressure typically seen after IRI. It is likely that ROS generated by NADPH oxidase directly affect vascular reactivity, thereby contributing to hypertension [42]. These findings are consistent with other studies that have also demonstrated the antihypertensive effects of APO, further supporting its role in modulating oxidative stress related vascular dysfunction [62,63]. While in vivo studies have demonstrated the protective effects of APO on renal function and blood pressure regulation following IRI, these findings are further supported by in vitro evidence. For instance, in a blood-perfused juxtamedullary nephron model, APO acutely restored afferent arteriolar vasoconstriction in response to increased perfusion pressure, effectively reversing ischemia–reperfusion-induced impairment of renal autoregulatory mechanisms [64].

In the majority of preclinical studies conducted to date, the effects of APO have been investigated under conditions in which the compound is administered either prophylactically or within a very short time frame following the induction of ischemia. While these approaches may demonstrate the pharmacological potential of APO, they do not adequately reflect the temporal limitations inherent to clinical practice, where therapeutic intervention is rarely feasible prior to or immediately after the ischemic event. Consequently, there is a critical need for preclinical models that initiate APO treatment in a delayed post-ischemic manner, in order to more accurately simulate the clinical setting and evaluate the translational relevance of its therapeutic efficacy.

### 4.2. The Effects of Apocynin in Nephrotoxic Acute Kidney Injury

Cisplatin is a widely used chemotherapeutic agent, but its clinical application is often limited by its dose-dependent nephrotoxicity [65], which manifests as AKI. Cisplatin, once converted into its highly reactive form, enhances the production of ROS and reacts with thiol-containing molecules like glutathione (GSH), leading to their depletion [66]. A reduction in cellular antioxidants can result in the buildup of endogenous ROS, which in turn activates signaling pathways that promote the death of renal tubular cells [5]. Different studies indicate that APO confers significant renoprotective effects in cisplatin-induced AKI through mechanisms involving attenuation of oxidative stress, suppression of inflammation, and inhibition of tubular apoptosis [45,46,47]. Chirino and colleagues [45] evaluated rats three days after a single intraperitoneal injection of cisplatin at a dose of 7.5 mg/kg. Apocynin was administered via drinking water at a concentration of 2 g/L, starting seven days prior to cisplatin administration and continuing for three days afterward. Treatment with APO significantly reduced the renal structural damage caused by cisplatin and improved kidney function markers. Specifically, it attenuated the elevations in blood urea nitrogen, serum creatinine, and urinary excretion of total protein, N-acetyl-β-D-glucosaminidase (NAG), and glutathione-S-transferase (GST) that were induced by cisplatin. The protective effect of APO was strongly linked to its ability to reduce cisplatin-induced oxidative and nitrosative stress. This was demonstrated by a decrease in renal malondialdehyde (MDA) levels, along with reduced expression of 3-nitrotyrosine (3-NT) and 4-hydroxynonenal (4-HNE), as assessed through immunohistochemical analysis. Similar effects were obtained in Wang et al. [46] and Meng et al. [47] studies, but with different protocols of APO application. Wang et al. [46] showed that a single pretreatment with APO (2 h before single i.p. cisplatin injection (20 mg/kg)) followed by two of the same additional daily doses (10 mg/kg i.p.) effectively mitigated cisplatin-induced kidney injury in male mice. This protective effect was associated with a reduction in cellular oxidative stress, caspase-3/7 activity, DNA fragmentation, cell death, and the expression of proinflammatory cytokines TNF-α and IL-1β. Moreover, the renoprotective effects of APO were dose-dependent, with higher doses offering greater protection against cisplatin-induced renal damage. They concluded that APO appears to exert its protective effects through a dual mechanism: first, by suppressing the activation of NOX2 and NOX4 in tubular epithelial cells and other intrinsic kidney cells in response to cisplatin; and second, by inhibiting NOX2 activity in infiltrating leukocytes, especially neutrophils. Meng et al. [47] also applied APO as pretreatment i.p. in a higher dose (100 mg/kg), one day before cisplatin. The cisplatin dose was the same. They showed that cisplatin markedly upregulated NOX4 expression in tubular epithelial cells and damaged kidney tissue. Their study also offers new evidence that inhibiting NOX4-driven ROS production reduces both necroptosis and apoptosis in tubular epithelial cells, thereby mitigating cisplatin-induced renal injury and associated inflammation.

Oxidative stress is believed to play a central role in the development of gentamicin (GM)-induced nephrotoxicity [67]. Despite its toxic effects, GM remains critical for treating severe infections caused by Gram-negative bacteria [68]. GM-induced kidney damage includes tubular necrosis, glomerular injury, renal apoptosis, and altered kidney function markers [69]. This damage results from GM accumulation in proximal tubule epithelial cells [70] leading to oxidative stress, ROS production, and reduced antioxidant enzyme activity [71]. GM also promotes proinflammatory cytokines (IL-1β, IL-6, IL-18, TNF-α) production [69] and activates transcription factors like NF-κB [72,73] and kidney injury markers (KIMs) [74], while suppressing protective ones such as Nrf2, AKT [75], and IL-10 [76]. Moreover, experimental studies have demonstrated that GM-induced nephrotoxicity is associated with an inflammatory response [77,78]. In this context, ROS are recognized for their role in initiating and propagating inflammatory responses [79], which may help explain the effectiveness of antioxidants in mitigating GM-induced renal injury [80,81]. Abdelrahman (2017) [48] showed that APO effectively alleviated the nephrotoxic and oxidative damage induced by GM, as evidenced by improved renal histology and significant reductions in 24 h urine output, serum creatinine, lactate dehydrogenase, blood urea nitrogen, urinary protein, and renal levels of malondialdehyde, CD95, and nitric oxide. Furthermore, APO treatment led to a notable increase in renal superoxide dismutase activity and creatinine clearance compared to the GM-treated group. These findings support the conclusion that APO mitigates GM-induced nephrotoxicity through its combined antioxidant, anti-inflammatory, and antiapoptotic properties.

In models of nephrotoxic AKI, the rationale for initiating APO treatment early is well-founded, as early administration maximizes its protective effects by mitigating oxidative stress before significant tissue damage occurs. This is particularly evident in experimental settings where APO is administered several days prior to nephrotoxin exposure. In theory, such a preventive approach could be translated to clinical practice, for example, by initiating treatment before administering nephrotoxic agents like gentamicin. However, in reality, this timing is often not practical, especially in patients with severe infections who require immediate antibiotic therapy. A delay of several days before initiating treatment is unrealistic in most clinical scenarios. Therefore, it is essential to explore whether APO remains effective when administered closer to or after the onset of nephrotoxin exposure, in order to better reflect clinically applicable treatment windows.

Contrast-induced nephropathy develops through three main but interconnected processes: reduced blood flow causing medullary ischemia, the generation of ROS, and the direct toxic effects of contrast agents on kidney tubule cells [82,83]. The outer region of the renal medulla naturally has low oxygen levels due to its limited oxygen supply combined with high metabolic demands, largely driven by active salt reabsorption in the thick ascending limbs of Henle’s loop. Contrast agents worsen this already low-oxygen environment by increasing metabolic activity and oxygen consumption, triggered by osmotic diuresis and greater salt delivery to the distal parts of the nephron. These hemodynamic changes lead to medullary hypoxia, which is followed by oxidative stress and subsequent tissue repair [50]. Ahmad et al. [50] explored the impact of APO on iomeprol-induced nephropathy in diabetic rats. Iomeprol is a nonionic, monomeric iodinated contrast agent used in medical imaging, particularly in CT scans and angiography. Rats were administered iomeprol (10 mL/kg i.v.) 30 min after saline administration. Their results showed that pretreatment with APO significantly reduced glomerular dysfunction, plasma sodium and potassium imbalances, markers of kidney injury (such as urinary alpha-GST and both plasma and urinary NGAL levels), kidney damage, nitrotyrosine formation and poly(ADP-ribose) polymerase activation (PARP, an enzyme involved in DNA repair), proinflammatory cytokine production, and cell apoptosis.

Cyclosporine (also known as cyclosporin A or CsA) is a powerful immunosuppressant that works by inhibiting the activation of T cells, specifically by blocking the transcription of cytokine genes [84]. While various factors contribute to the pathophysiology of CsA-induced nephrotoxicity, oxidative stress plays a pivotal role in both its initiation and progression. CsA-induced kidney damage was halted by sitagliptin and hesperidin via increasing Nrf2 and suppressing TNF-α, NF-κB, and Bax [85]. This drug is commonly used in solid organ transplants. Nonetheless, the use of CsA is linked to several adverse effects, most notably kidney toxicity and high blood pressure [86]. It has been proposed that NOX4 is the main contributor to superoxide (O_2_^−^) production in renal cortex homogenates [87]. Under normal physiological conditions, O_2_^−^ exists at low levels because the enzyme superoxide dismutase rapidly converts it into hydrogen peroxide (H_2_O_2_). Although NOX4 normally exhibits low constitutive activity, its activity can increase significantly in response to cytokines [88] and growth factors [89]. NOX4 is easily distinguished from other NADPH oxidase isoforms in the NOX protein family due to its notably high expression in renal tissues [90]. Tan et al.’s [49] research indicated that kidney injury in the CsA model is linked to elevated NADPH oxidase activity, leading to increased superoxide (O_2_^−^) production and contributing to both renal damage and hypertension. Preventive treatment with APO over a 14-day period helped maintain normal blood pressure, improved kidney function, and effectively prevented CsA-induced tissue injury. While acute nephrotoxicity caused by cyclosporine A (CsA) involves reversible hemodynamic changes, chronic CsA-induced nephrotoxicity is marked by sustained and often irreversible alterations, including persistent vasoconstriction of glomerular afferent arterioles, leading to reduced renal perfusion, decreased GFR, and the development of hypertension [91]. Since both acute and chronic forms of CsA nephrotoxicity share common underlying mechanisms, particularly increased superoxide production and reduced nitric oxide (NO) availability due to NADPH oxidase activation, it is reasonable to propose that APO may also exert protective effects in chronic nephrotoxicity [92,93]. By targeting oxidative pathways implicated in long-term renal damage, APO could help preserve renal function and mitigate hypertension during prolonged CsA treatment. In this context, a rat model demonstrated that co-treatment with APO effectively alleviated the adverse effects of CsA by reducing oxidative stress and restoring NO bioavailability [94]. These findings underscore APO’s potential as a protective agent against CsA-induced chronic renal and vascular side effects by correcting oxidative imbalance and preserving endothelial, vascular, and renal function.

Acrylamide (ACR) is a toxic compound capable of triggering and promoting the progression of AKI [95]. ACR is a chemical substance commonly used in the manufacturing of various industrial and consumer products [96]. Apoptosis, oxidative stress, and inflammation are the basic mechanisms through which acrylamide exerts its toxic effects [97]. Ageena and colleagues [51] showed that the levels of renal function biomarkers, serum urea, creatinine, and uric acid were significantly elevated after ACR exposure, accompanied by disturbances in electrolyte balance (Na^+^, K^+^, and Mg^2+^). However, treatment with APO led to a marked reduction in these biomarkers and helped restore normal electrolyte levels. In addition, APO treatment decreased renal KIM-1 expression. A decrease in KIM-1 expression may reflect a reduction in kidney injury [98]. In this study, ACR administration led to upregulation of the NLRP3 inflammasome and proinflammatory cytokines. Notably, treatment with APO protected rats from ACR-induced AKI by downregulating NLRP3 expression and inhibiting the assembly and activation of the inflammasome complex. This intervention reduced IL-1β release and inflammation by modulating key components of the NLRP3 pathway, including NLRP3, Gasdermin D, IL-1β, and caspase-1. Moreover, APO has been shown to suppress NLRP3 inflammasome expression and mitigate renal fibrotic changes [99].

The therapeutic effects of APO in preclinical models appear to be highly dependent on dose, treatment duration, and the route and timing of administration. Various studies have employed a wide range of dosing regimens, often without a standardized protocol, which complicates the interpretation and comparison of results. While low-to-moderate doses have demonstrated antioxidant and anti-inflammatory properties, the efficacy and safety profile at higher doses remain less well-defined. In most studies, APO is administered systemically, typically via intraperitoneal or intravenous injection, and treatment is often initiated prior to or shortly after injury induction. The duration of treatment varies substantially across models, ranging from a single dose to repeated administration over several days. However, the optimal therapeutic window and treatment duration for sustained protective effects without adverse outcomes have yet to be clearly established. To facilitate clinical translation, future studies should aim to systematically evaluate the dose–response relationship, define the minimal effective dose, and investigate the impact of delayed or prolonged administration on therapeutic efficacy and safety.

### 4.3. The Effects of Apocynin in Other Types of Acute Kidney Injury

Mittwede with coworkers [52] evaluated the role of oxidative stress in orthopedic trauma-induced AKI in obese Zucker (OZ) rats. After trauma, obese Zucker (OZ) rats showed a significant decline in glomerular filtration rate. They also had marked increases in plasma creatinine levels, urinary KIM-1, and albumin excretion. Furthermore, oxidative stress was elevated in OZ rats, as indicated by higher renal NADPH oxidase activity and increased urinary levels of lipid peroxidation products (thiobarbituric acid-reactive substances). At the same time, renal superoxide dismutase activity was significantly reduced in these rats. Treatment with APO alleviates AKI in OZ rats and decreases both systemic and renal inflammation. Similarly to previous findings [100], this study demonstrated that APO, when used as an antioxidant, was effective even with short-term administration.

Acute hypertriglyceridemic pancreatitis (HTGP) tends to present more severely and is more frequently associated with injury of extrapancreatic organs [54]. During the onset and progression of HTGP, serum triglycerides can be broken down by pancreatic lipase into large amounts of free fatty acids (FFAs), which play a key role in promoting oxidative stress [101]. In addition, studies have shown that renal injury associated with acute pancreatitis (AP) is primarily due to oxidative stress, which leads to the loss of barrier integrity and dysfunction of renal tubular epithelial cells [102]. At the same time, oxidative stress can stimulate the production of numerous inflammatory mediators, which increase capillary permeability and further worsen renal tissue damage [103]. In renal tissue, NOX4 and NOX2 are widely expressed in glomerular cells, renal tubular cells, and interstitial cells. Abnormal expression levels of these NADPH oxidases are closely associated with renal injury [104]. Apocynin reduces the production of ROS in the kidney by inhibiting NOX4. This suppression leads to decreased activation of NF-κB via the PI3K/Akt signaling pathway, resulting in lower release of inflammatory cytokines and protection against renal injury in rats with acute hypertriglyceridemic pancreatitis (HTGP) [53].

In addition, there is a clear need for preclinical and clinical studies evaluating the therapeutic potential of APO in sepsis-associated AKI. Septic AKI is a complex and multifactorial condition characterized by hemodynamic alterations, inflammation, endothelial dysfunction, and oxidative stress [105]. Among these, excessive production of ROS plays a critical role in the pathogenesis of renal injury [106]. By inhibiting NOX-derived ROS, APO may offer protective effects in this setting. However, despite its theoretical benefits and evidence of efficacy in other models of renal and systemic inflammation, direct experimental or clinical evidence supporting the use of APO in septic AKI is currently lacking. Furthermore, the dual role of ROS in septic AKI [106], as both mediators of host defense and contributors to tissue injury, raises concerns regarding the timing and extent of ROS inhibition. While early administration of APO may prevent excessive oxidative damage, indiscriminate or delayed suppression of ROS could interfere with antimicrobial responses. Thus, further studies are needed to better define its therapeutic window, optimal dosing, and potential interactions with the host immune response in the context of sepsis.

It is also important to emphasize that studies using NADPH oxidase knockout (NOX-KO) mice, particularly NOX2 and NOX4-deficient strains, have provided critical insights into the pathophysiological roles of ROS in AKI. Nlandu-Khodo et al. [107], demonstrated that NOX4 deficiency exacerbates tubular cell death following IRI, suggesting that NOX4 may play a protective role under certain stress conditions. In this model, NOX4 knockout mice exhibited significantly increased tubular apoptosis, elevated serum creatinine levels, and worsened renal function compared to wild-type controls. These findings challenge the prevailing view of NOX4 as purely deleterious. Consequently, these results underscore the complexity of targeting NADPH oxidase isoforms therapeutically. In the context of pharmacological inhibitors such as APO, which non-selectively inhibits NOX-derived ROS, there is a potential risk of disrupting protective redox signaling, particularly when NOX4 activity contributes to renal cell adaptation or survival. Thus, isoform- and context-specific approaches are critical for effective and safe modulation of oxidative stress in AKI. Conversely, NOX2, predominantly expressed in infiltrating immune cells, contributes to the inflammatory response in AKI. In a cisplatin-induced AKI model, NOX2 knockout mice showed improved renal function, reduced tubular damage, and lower expression of injury markers like KIM-1, IL-6, and IL-1α. Importantly, NOX2 deficiency mitigated neutrophil infiltration via reduced ICAM-1 and CXCL1 expression [108]. In IRI models and kidney transplantation settings, NOX2-KO mice exhibited decreased inflammation, oxidative stress, α-SMA-mediated fibrosis, and oxidative lipid peroxidation, underlining its detrimental role in AKI progression [109]. These findings highlight the complex and potentially distinct roles of individual NOX isoforms, underscoring the need for isoform-specific targeting rather than broad-spectrum NOX inhibition.

While animal experimental studies have demonstrated APO’s renoprotective effects in both ischemic and nephrotoxic AKI, cell culture models have been instrumental in identifying the cellular targets and molecular pathways involved, particularly its inhibition of NADPH oxidase activity and modulation of ROS signaling. Collectively, the cell culture studies by Bhatt et al. [110], Shen et al. [111], and Lu et al. [112] provide compelling evidence for the antioxidant and anti-inflammatory effects of APO at the cellular level. Bhatt et al. (2017) [110] demonstrated that APO reduces inflammatory mediators in LPS-stimulated renal mesangial cells by inhibiting MAPK signaling and activating the HO-1/Nrf2 pathway. Shen et al. (2016) [111] found that APO attenuates endothelial activation by blocking indoxyl sulfate-induced ROS generation and subsequent MAPK, NF-κB, and AP-1 activation. Lu et al. (2020) [112] further showed that in human proximal tubular cells, APO mitigates salusin-β-induced oxidative stress and apoptosis through inhibition of the PKC/ROS/p53 signaling pathway. Despite being limited to in vitro models, these studies consistently highlight APO’s capacity to counteract oxidative stress-mediated injury in renal and vascular cell types, supporting its potential for therapeutic development.

At the end of this discussion, we would also like to point out that, in addition to NADPH oxidase, several other sources contribute to ROS generation during AKI, including mitochondrial dysfunction [113], xanthine oxidase activation [114], uncoupled nitric oxide synthase (NOS) [115], and endoplasmic reticulum stress [116]. While APO is primarily known as an NADPH oxidase inhibitor, its antioxidant properties may also exert indirect effects on other ROS generating pathways by lowering the overall oxidative burden and suppressing inflammatory signaling. However, since APO does not directly target mitochondrial or other enzymatic sources of ROS, there are concerns about whether inhibition of a single pathway is sufficient to fully mitigate oxidative damage in AKI. Therefore, future studies should explore the potential of combining APO with agents that target alternative ROS sources, which may enhance its protective efficacy across different AKI models.

Furthermore, while NADPH oxidase inhibition represents a promising therapeutic approach for reducing oxidative stress in AKI, there are important considerations and potential complications associated with targeting this enzyme system. NADPH oxidases (NOX isoforms) are not only involved in pathological ROS production but also play essential roles in normal cellular signaling, immune defense, vascular tone regulation, and host–microbe interactions [117]. Broad or non-selective inhibition may therefore impair physiological ROS functions, potentially leading to unintended immunosuppression, reduced antimicrobial activity, or vascular dysregulation. Additionally, the heterogeneity of NOX isoforms (e.g., NOX1, NOX2, NOX4, NOX5) across different tissues and cell types complicates the development of selective inhibitors, raising the risk of off-target effects. Some inhibitors, including APO, have been reported to exhibit inconsistent efficacy depending on the redox state of the cell or the presence of peroxidase enzymes, which are required for its activation. These limitations highlight the need for further research into isoform-specific NADPH oxidase inhibitors, careful dosing strategies, and combination therapies that can achieve redox balance without compromising essential physiological functions.

Despite APO’s promise as an NADPH oxidase inhibitor, its reliance on MPO-mediated activation and its potentially limited efficacy against non-leukocyte NOX isoforms raise concerns about its consistency and selectivity in vivo [118]. Moreover, high levels of reduced thiols in some tissues may inactivate APO’s reactive metabolites, further limiting its action [25]. These factors highlight the need for further studies to confirm the efficacy and selectivity of APO, particularly in diverse in vivo settings, and to better define its therapeutic potential in clinically relevant models.

## 5. Conclusions

Experimental preclinical studies have consistently demonstrated that APO provides protective effects in models of ischemic and nephrotoxic kidney injury, primarily through inhibition of NADPH oxidase-mediated oxidative stress. In renal ischemia–reperfusion injury and drug-induced nephrotoxicity models, APO has been shown to reduce oxidative damage, suppress inflammation, and preserve renal structure and function. These findings underscore its potential as a therapeutic agent for mitigating kidney injury in acute ischemic conditions or exposure to nephrotoxic agents. However, despite these promising results, a significant limitation remains: there is a lack of comprehensive data regarding the optimal dosing strategies, treatment duration, and administration protocols. Moreover, current evidence is largely confined to preclinical settings, with no clinical studies conducted to date. This highlights the urgent need for further research to clarify APO’s pharmacodynamics, refine its therapeutic application, and assess its translational relevance and safety in human pathologies. 

## Figures and Tables

**Table 1:** Effects of apocynin on postischemic acute kidney injury.

Author	Experimental Protocol	Apocynin Protocol	Main Findings
Altintas et al., 2013 [37]	Ischaemia (60 min)-reperfusion Injury induced in Wistar albino rats	30 min before ischemia, 20 mg/kg i.p. (one group) and 30 min before reperfusion, 20 mg/kg i.p. (other group)	APO applied 30 min before ischemia significantly decreased serum levels of creatinine and urea, decreased lipid peroxidation and MPO activity, increased GPx activity in kidney tissue, and improved renal morphology (evaluated by semiquantitative analysis). APO applied 30 min after ischemia also significantly decreased serum creatinine and urea, increased only GPx activity in kidney, improved renal morphology, and decreased caspase-3 positive cells in tubular immunostaining.
Li and Wang, 2015 [38]	Ischaemia (45 min)-reperfusion Injury induced in Sprague—Dawley rats	30 min before reperfusion, 50 mg/kg i.p.	APO significantly decreased creatinine and urea serum levels, decreased lipid peroxidation and iNOS activity, improved SOD activity, increased NO levels, decreased TNF-α levels, decreased TLR4 and NF-κB, and increased SHP-1 expression in kidney tissue.
Choi et al., 2015 [39]	Ischaemia (30 min)-reperfusion Injury induced in Sprague—Dawley rats	1 h before ischemia, 20 mg/kg i.p.	APO significantly decreased serum levels of creatinine and urea, decreased lipid peroxidation, and improved renal morphology (significant recovery in the tubular cells).
Hu et al., 2016 [40]	Ischaemia (45 min)-reperfusion Injury induced in Sprague—Dawley rats	30 min before ischemia, 50 mg/kg i.p.	APO decreased serum creatinine and urea levels, increased serum and kidney tissue zinc levels, increased metallothionen expression, decreased lipid peroxidation and increased SOD activity, increased IL-4 and IL-10, and decreased IL-6 and TNF-α levels in kidney tissue.
Kovacevic et al., 2020 [41]	Ischemia (45 min)-reperfusion injury induced in Spontaneously hypertensive rats	5 min before reperfusion, 40 mg/kg i.v.	APO improved renal hemodynamics (increased RBF and decreased RVR), decreased creatinine, urea, and phosphate levels in plasma, and improved renal morphology (moderately intensive tubular necrosis, reduced tubular dilatation, and a smaller number of PAS-positive casts).
Lima et al., 2021 [42]	Ischaemia (45 min)-reperfusion Injury induced in Wistar albino rats	24 h before and after ischemia, 100 mg/kg in drinking water (acute treatment), 24 h after ischemia, 100 mg/kg in drinking water, 4 weeks (chronic treatment)	APO reduced creatinine and urea serum levels 24 h after reperfusion. Acute APO treatment decreased lipid peroxidation, superoxide anion production, NOX oxidase activity, ouabain-sensitive Na^+^-K^+^-ATPase activity, AT1 and AT2 receptor expression and increased furosemide-sensitive, ouabain-resistant Na^+^-K^+^-ATPase activity, ACE 1 and ACE 2 activity, and AT2 receptor expression in kidney tissue 4 weeks after AKI induction. Chronic APO treatment also decreased lipid peroxidation, superoxide anion production, NOX oxidase activity, and AT1 receptor expression, and increased furosemide-sensitive, ouabain-resistant Na^+^-K^+^-ATPase activity, AT2 receptor expression, and ACE 1 and PKCλ activity in kidney tissue 4 weeks after AKI induction.
Kovacevic et al., 2021 [43]	Ischaemia (45 min)-reperfusion injury induced in Spontaneously hypertensive rats	5 min before reperfusion, 40 mg/kg i.v. 2 sessions	APO improved clearances of urea, creatinine, and phosphate, decreased expression of 4-HNE and NGAL, and provoked different HO-1 immunohistochemical expression patterns in kidney tissue.
Revesz et al., 2024 [44]	Ischaemia (15, 20 or 30 min)-reperfusion Injury induced in C57BL/6 mice	15 min before (i.v.), immediately after (i.p.), and 120 min after (i.p.) ischemia, 10 mg/kg i.p.	APO decreased urea and NGAL levels in plasma, increased NRF2, HO-1, and GPx3, and decreased NGAL, TNF-α, and F4/80 mRNA expression in kidney tissue and improved renal morphology (decreased ATN score) after mild (15 min) ischemia. APO did not have beneficial effects after moderate (20 min) or severe (30 min) ischemia.

APO—Apocynin; MPO—Myeloperoxidase; GPx—Glutathione peroxidase; iNOS—Inducible nitric oxide synthase; SOD—Superoxide dismutase; NO—Nitric oxide; TNF-α—Tumor necrosis factor alpha; TLR4—Tool-like receptor 4; NF-κB—Nuclear factor kappa-light-chain-enhancer of activated B cells; SHP-1—Src homology-2-domain-containing phosphatase-1; IL—Interleukin; RBF—Renal blood flow; RVR—Renal vascular resistance; AT1—Type I angiotensin II; AT2—type II angiotensin II; ACE 1—Angiotensin-converting enzyme—isoform 1; ACE 2—Angiotensin-converting enzyme-isoform 2; PKC—Protein kinase C; 4-HNE—4-hydroxy-2-nonenal; NGAL—Neutrophil gelatinase-associated lipocalin; HO-1—Heme oxygenase-1; NRF2—Nuclear factor erythroid 2-related factor 2; ATN score—Acute tubular necrosis score.

**Table 2:** Effects of apocynin on acute kidney injury caused by other etiologies.

Author	Experimental Protocol	Apocynin Protocol	Main Findings
Chirino et al., 2008 [45]	Cisplatin-induced nephrotoxicity in Wistar albino rats	7 days before and 3 days after single i.p. cisplatin injection (7.5 mg/kg), 2 g/L in drinking water.	APO significantly decreased serum levels of creatinine and urea, proteinuria and urinary GST and NAG excretion, decreased lipid peroxidation, immunohistochemical expression of 4-HNE and 3-NT in kidney tissue, and improved renal morphology (nearly regular morphology of epithelial tubular cells with significant reduction of cast formation of 85% in renal cortex and of 98% in renal medulla).
Wang et al., 2015 [46]	Cisplatin-induced nephrotoxicity in C56BL/6 mice	2 h before and 2 days after single i.p. cisplatin injection (20 mg/kg), 10 mg/kg i.p.	APO significantly decreased creatinine and urea in serum, improved kidney morphology (necrosis, protein cast, vacuolation, and desquamation of epithelial cells in the renal tubules was significantly attenuated, up to 40%), decreased oxidative stress (3-NT, 4-HNE), NOX activity, decreased inflammation (decreased TNF-α, IL-1β expression and MPO activity), and decreased apoptosis (decreased cleaved caspase 3, DNA fragmentation, TUNEL + cells) in kidney tissue.
Meng et al., 2017 [47]	Cisplatin-induced nephrotoxicity in mice	1 day before single i.p. cisplatin injection (20 mg/kg), 100 mg/kg i.p.	APO significantly decreased creatinine and urea in serum, decreased lipid peroxidation, NOX 4 protein expression, KIM-1 expression, decreased inflammation (decreased TNF-α, IL-1β and IL-6 expression), decreased RIPK1, RIPK3, P-MLKL and cleaved caspase 3 in kidney tissue, and improved renal morphology (tubular necrosis, cast formation and tubular dilatation significantly reduced).
Abdelrahman, 2017 [48]	Gentamicin-induced nephrotoxicity in Wistar albino rats	7 days before and 7 days along with i.p. gentamicin injection (100 mg/kg, 7 days), 10 mg/kg i.p.	APO decreased serum creatinine and urea levels, increased creatinine clarence, decreased proteinuria, decreased lipid peroxidation and NO content, increased SOD activity, decreased CD95 in kidney tissue, and improved renal morphology (normal renal tubular epithelium lining renal tubules).
Tan et al., 2020 [49]	Cyclosporine A-induced nephrotoxicity in Wistar-Kyoto rats	14 days along with cyclosporine-A administration (25 mg/kg/day, via gavage), 2.5 mmol/L/day orally	APO decreased plasma creatinine and urea, increased creatinine clarence, decreased urine output, fractional excretion of sodium, urinary sodium/potassium ratio, decreased BUN and proteinuria, improved renal cortical blood perfusion, decreased lipid peroxidation, increased SOD activity and total antioxidant capacity, decreased NOX 4 mRNA expression in kidney tissue, and improved renal morphology (no severe renal tubular ischemia, no abscesses in renal interstitial area, normal glomerular apparatus, no neutrophils).
Ahmad et al., 2012 [50]	Contrast-induced nephrotoxicity in diabetic Wistar albino rats	30 min before i.v. iomeprol injection (10 ml/kg), 5 mg/kg i.v.	APO decreased plasma creatinine and urea, increased creatinine clarence, decreased plasma and urinary NGAL, decreased urinary αGST, decreased immunohistochemical 3-NT, TNF-α, IL-1β expression, PARP activation and number of apoptotic calls or fragments in kidney tissue, and improved renal morphology (significantly decreased histological score).
Ageena et al., 2021 [51]	Acrylamide-induced nephrotoxicity in Wistar albino rats	10 days along with acrylamide administration (40 mg/kg/day, i.p.), 100 mg/kg orally	APO decreased serum creatinine, urea, uric acid, potassium, magnesium, and KIM-1, decreased lipid peroxidation, increased GSH and SOD levels, increased NRF-2 and HO-1 expression, decreased TNF-α, caspase 1, IL-1β, GSDMD, ASC, and NLRP3 expression in kidney tissue, with mild improvement in renal morphology (moderate damage to proximal and distal convoluted tubules, moderate hyperemia of the capillary tufts and blood vessels, and moderate interstitial hemorrhage).
Mittwede et al., 2015 [52]	Orthopedic trauma-induced acute kidney injury in obese Zucker rats	Immediately after trauma, 50 mg/kg, i.p., and during next 24 h in drinking water, 2 mmol.	APO decreased creatinine levels in plasma, improved GFR, decreased urinary KIM-1, albumin excretion, and urinary lipid peroxidation, decreased NOX activity and IL-6 in kidney tissue.
Yang et al., 2020 [53]	Acute hypertriglyceridemic pancreatitis- related acute kidney injury induced in Sprague–Dawley rats	30 min before induction, 50 mg/kg, subcutaneously	APO significantly decreased creatinine and urea serum levels, decreased NOX2, NOX4 expression, ROS levels, TNF-α, NF-κβ, GSK-3β and increased p-AKT expression, reduced MPO and CD68 positive cells, decreased apoptotic index, and improved renal morphology (histopathological score was significantly reduced).

APO—Apocynin; GST—Glutathione S-transferase; NAG—N-acetyl-beta-D-glucosaminidase; 4-HNE—4-hydroxy-2-nonenal; 3-NT—3-Nitrotyrosine; NOX—NADPH oxidase; TNF-α—Tumor necrosis factor alpha; IL—Interleukin; MPO—Myeloperoxidase; TUNEL—Terminal deoxynucleotidyl transferase dUTP nick end labeling; KIM-1—Kidney injury molecule-1; RIPK—Receptor interacting serine/threonine-protein kinase; P-MLKL—Phosphorylated-Mixed lineage kinase domain like pseudokinase; NO—Nitric oxide; SOD—Superoxid dismutase; CD95—Cluster of differentiation 95; BUN—Blood urea nitrogen; NGAL—Neutrophil gelatinase-associated lipocalin; PARP—Poly (ADP-ribose) polymerase; GSH—Gluthatione; NRF-2—Nuclear factor erythroid 2-related factor 2; HO-1—Heme oxygenase-1; GSDMD—Gasdermid D; ASC—Apoptosis associated speck-like protein, NLRP3—nucleotide-binding domain, leucine rich containing family, pyrin domain-containing-3; GFR—Glomerular filtration rate; NF-κB—Nuclear factor kappa-light-chain-enhancer of activated B cells; CD68—Cluster of differentiation 68; AKT—Protein kinase B; GSK-3β—Glycogen synthase kinase-3 beta.
